# Array-based gene expression, CGH and tissue data defines a 12q24 gain in neuroblastic tumors with prognostic implication

**DOI:** 10.1186/1471-2407-10-181

**Published:** 2010-05-05

**Authors:** Maija Wolf, Miikka Korja, Ritva Karhu, Henrik Edgren, Sami Kilpinen, Kalle Ojala, Spyro Mousses, Anne Kallioniemi, Hannu Haapasalo

**Affiliations:** 1 Institute for Molecular Medicine Finland (FIMM), University of Helsinki, Tukholmankatu 8, FIN-00290, Helsinki, Finland; 2 Medical Biotechnology, VTT Technical Research Centre of Finland and University of Turku, Itäinen Pitkäkatu 4C, FIN-20520 Turku, Finland; 3 Department of Medical Biochemistry and Molecular Biology, University of Turku, FIN-20520 Turku, Finland; 4 Department of Neurosurgery, Helsinki University Central Hospital, FIN-00029 HUS, Finland; 5 Laboratory of Cancer Genetics, Tampere University Hospital and Institute of Medical Technology, University of Tampere, FIN-33520 Tampere, Finland; 6 Pharmaceutical Genomics Division, The Translational Genomics Research Institute, Scottsdale, Arizona 85259, USA; 7 Department of Pathology, Tampere University Hospital, FIN-33521 Tampere, Finland

## Abstract

**Background:**

Neuroblastoma has successfully served as a model system for the identification of neuroectoderm-derived oncogenes. However, in spite of various efforts, only a few clinically useful prognostic markers have been found. Here, we present a framework, which integrates DNA, RNA and tissue data to identify and prioritize genetic events that represent clinically relevant new therapeutic targets and prognostic biomarkers for neuroblastoma.

**Methods:**

A single-gene resolution aCGH profiling was integrated with microarray-based gene expression profiling data to distinguish genetic copy number alterations that were strongly associated with transcriptional changes in two neuroblastoma cell lines. FISH analysis using a hotspot tumor tissue microarray of 37 paraffin-embedded neuroblastoma samples and *in silico *data mining for gene expression information obtained from previously published studies including up to 445 healthy nervous system samples and 123 neuroblastoma samples were used to evaluate the clinical significance and transcriptional consequences of the detected alterations and to identify subsequently activated gene(s).

**Results:**

In addition to the anticipated high-level amplification and subsequent overexpression of *MYCN, MEIS1, CDK4 *and *MDM2 *oncogenes, the aCGH analysis revealed numerous other genetic alterations, including microamplifications at 2p and 12q24.11. Most interestingly, we identified and investigated the clinical relevance of a previously poorly characterized amplicon at 12q24.31. FISH analysis showed low-level gain of 12q24.31 in 14 of 33 (42%) neuroblastomas. Patients with the low-level gain had an intermediate prognosis in comparison to patients with *MYCN *amplification (poor prognosis) and to those with no *MYCN *amplification or 12q24.31 gain (good prognosis) (*P *= 0.001). Using the *in silico *data mining approach, we identified elevated expression of five genes located at the 12q24.31 amplicon in neuroblastoma (*DIABLO, ZCCHC8, RSRC2, KNTC1 *and *MPHOSPH9*). Among these, *DIABLO *showed the strongest activation suggesting a putative role in neuroblastoma progression.

**Conclusions:**

The presented systematic and rapid framework, which integrates aCGH, gene expression and tissue data to obtain novel targets and biomarkers for cancer, identified a low-level gain of the 12q24.31 as a potential new biomarker for neuroblastoma progression. Furthermore, results of *in silico *data mining suggest a new neuroblastoma target gene, *DIABLO*, within this region, whose functional and therapeutic role remains to be elucidated in follow-up studies.

## Background

Cancer is a complex disease caused by mechanisms that disrupt cell homeostasis in many levels. Such mechanisms include aberrations affecting gene copy numbers, leading to altered gene expression and deregulation of critical signalling pathways. Neuroblastoma is an early childhood malignancy arising from undifferentiated neuroectodermal cells derived from the neural crest. These neural crest precursor cells are committed to differentiate into cells that make up sympathetic ganglia or the adrenal medulla. The most well known genetic alteration in neuroblastoma is the amplification of the *MYC*-related oncogene (*MYCN*) [1,2], which is still the only prognostically significant oncogene amplification in neuroblastoma [3,4]. Despite numerous other genetic alterations in neuroblastoma, such as deletions/losses/gains of 1p36, 1q, 2p13-p14, 3p21, 3p26, 3q24-p26, 4q33-q35, 6p11-p22, 11q23, 12q, 14q32, 17q and 19q [5-17], none of these alterations has been consistently shown to have a definite independent value in treatment stratifications. Unfortunately, the main genetic alteration, *MYCN *amplification, does not explain the poor outcome of all neuroblastoma patients, suggesting that additional biomarkers of disease progression are still needed.

Here, we present a systematic and rapid genomics data analysis framework, which integrates DNA, RNA and tissue data to identify clinically relevant biomarkers for neuroblastoma. In more detail, high-resolution aCGH was utilized to identify novel genetic alterations in two neuroblastoma cell lines, NGP and IMR-32. Through the integration of gene copy number and gene expression data, the impact of copy number changes on expression levels was determined. Fluorescence *in situ *hybridization (FISH) on a tissue microarray (TMA) format was used to assess the clinical significance of the identified copy number increase at 12q24.31 in neuroblastoma patients. Finally, we used *in silico *data mining of publicly available transcriptomics data, to evaluate the transcriptional consequences of the detected 12q24.31 alteration and to identify subsequently activated gene(s).

## Methods

### Neuroblastoma cell cultures and sample preparation

NGP and IMR-32 neuroblastoma cells were cultured in RPMI 1640 medium supplemented with 10% fetal bovine serum (FBS) and 2 mM L-glutamine, and Minimum Essential Medium supplemented with 10% FBS, 2 mM L-glutamine, 1% non-essential amino acids and 1% sodium pyruvate, respectively. mRNA was isolated from the samples using FastTrack 2.0 mRNA isolation kit (Invitrogen, Carlsbad, CA). Genomic DNAs were obtained from the same samples by swirling a glass rod in the cell lysate, followed by standard phenol-chloroform purification.

### Oligonucleotide array-based comparative genomic hybridization

A 95K high-resolution oligonucleotide array (Agilent Technologies, Palo Alto, CA) was used for the detection of copy number changes in NGP and IMR-32 cell lines. Normal male DNA was used as a reference for both cell lines (Cat. # G1471, Promega, Madison, WI). Sample processing and hybridization was performed according to the August 2005 (version 2) protocol (Agilent Technologies), with minor modifications. Briefly, 10 μg of genomic DNA was digested overnight with *Alu*I and *Rsa*I (Life Technologies, Inc., Rockville, MD). Digested DNA samples were subjected to standard phenol-chloroform purification. 4 μg of digested tumor DNA and reference DNA were labelled with Cy5-dUTP and Cy3-dUTP (Perkin-Elmer, Wellesley, MA), respectively, in a random priming reaction using a BioPrime Array CGH Genomic Labelling Module (Invitrogen, Carlsbad, CA.). After labelling, tumor DNA and reference DNA samples were pooled, cleaned and hybridization cocktails were prepared according to the protocol. Hybridization and washes were also performed according to the protocol. A laser confocal scanner (Agilent Technologies) was used to obtain signal intensities from targets and Feature Extraction software (version 8.1.1.1, Agilent Technologies) was used in image analysis with settings recommended by the manufacturer (44K_CGH_0605). The CGH Analytics software was used for analysis of aCGH data (version 3.2.32, Agilent Technologies). Control hybridizations (male vs. male, male vs. female) were used to estimate the baseline variation in the hybridizations, and hybridization quality metrics provided by CGH Analytics were evaluated to ensure good data quality.

Amplifications and losses were defined at loci with log2 copy number ratios >2 (amplification) or <0.5 (loss). Loci with log2 copy number ratio > 3.5 were considered as high-level amplifications.

### Gene expression analyses

Gene expression levels from NGP and IMR-32 cell lines were measured using Affymetrix GeneChip Human Genome U133 Plus 2.0 arrays (Affymetrix, Santa Clara, CA). In addition, a pooled sample consisting of 16 different cancer cell lines was analyzed as a reference sample. Sample processing and labelling were performed according to the protocol provided by the Affymetrix. Briefly, 3 μg of mRNA was used for one-cycle cDNA synthesis using a T7-oligo(dT) promoter primer, followed by RNase H-mediated second-strand cDNA synthesis and *in vitro *transcription reaction with biotinylated ribonucleotide analogs. Biotin-labelled target cRNAs were fragmented and the quality of labelling procedures was assessed with GeneChip Test3 arrays. Hybridizations to U133 Plus 2.0 arrays were performed for 16 hours at 45°C, followed by automated array washing and staining procedures. Arrays were scanned immediately after staining using a GeneChip scanner (Affymetrix). Basic GeneChip array analysis was performed for the obtained image file. Data preprocessing was done using the MAS5 algorithm implemented in the Bioconductor package "affy" [18].

Data from both aCGH and gene expression analysis has been deposited in NCBI's Gene Expression Omnibus (GEO) and are accessible through Series record GSE18144.

### Integration of the aCGH and gene expression data

Expression ratios for the NGP and IMR32 cell lines were calculated as the log2 ratio of the cell line divided by the reference pool hybridization. A gene was considered over- or underexpressed if its expression ratio was within the top or bottom 7% of ratios in that sample, respectively. Expression and copy number data was integrated as follows: Affymetrix probe sets were mapped to Ensembl gene IDs or the base pair position given by Affymetrix, if no matching Ensembl gene id was found. A copy number ratio for each probe set was then calculated as the median of CGH array oligos located within 50 kb of the probe set's genomic position. In order to investigate the impact of copy number on expression ratios, genes were divided into bins based on their copy number and the frequency of over- and underexpressed genes in each bin was calculated.

### Hotspot FISH on a neuroblastoma TMA

For the TMA construction, a total of 37 archival paraffin-embedded neuroblastomas were obtained from the Turku University Hospital and the Tampere University Hospital, Finland. These tumor samples represented a vast proportion of all neuroblastomas diagnosed in these two hospitals during 1967-2001. All the tumors were immunohistochemically stained and microscopically re-evaluated by two experienced pathologists. For histological typing and grading (excluding one intracranial tumor and two cytotoxically pretreated tumors), the INPC definition was applied [19]. The 34 classified tumors (31 primary tumors and three metastases) comprised 31 neuroblastomas (14 undifferentiated, eight poorly differentiated, and nine differentiating), two ganglioneuroblastomas (one nodular, one intermixed) and one ganglioneuroma. Of these 34 cases, nine (26%) were with favourable, and 25 (74%) with unfavourable histology.

The most proliferative areas of the tumors, *i.e*. hotspots, were selected to be sampled for the TMA using core biopsies with a diameter of 2 mm. More detailed TMA characteristics and data on the construction of hotspot neuroblastoma TMA have been described previously [20]. Twelve bacterial artificial chromosome (BAC) clones from 12q13-q14 (RP11-846E20, RP11-66N19), 12q15 (RP11-1024C4, RP11-77H17), and 12q24.31 (RP11-44F24, RP11-87C12, RP11-94C5, RP11-512 M8, RP11-152E17, RP11-679G17, RP11-1059L20, and RP11-486O12) were labelled with digoxigenin-11-dUTP (Roche Applied Science, Basel, Switzerland) using random priming. A spectrum Green labelled chromosome 12 specific centromere probe (Vysis Inc. Downers Grove, IL) was used as a reference. FISH on the neuroblastoma TMA containing 37 samples was performed as described [21], except that the fixation of the slides was performed using 7% formalin for 10 min. The BAC probes were detected with anti-digoxigenin-Rhodamine (Roche Applied Science), and nuclei counterstained with 0.1 M 4',6-diamidino-2-phenylindole. The fluorescence signals were scored from non-overlapping nuclei using Olympus BX50 epifluorescence microscope (Tokyo, Japan). The entire tissue area was evaluated and 20-60 representative non-overlapping nuclei were scored. A 1.5-fold increase in the test probe copy number relative to the chromosome 12 centromere was considered as gain in copy number. *MYCN *amplification status was assessed using the chromogenic *in situ *hybridization technique as described [20].

Differences between groups in categorical data were analyzed by means of the Pearson chi-square test. Overall survival analysis was computed by means of the Kaplan-Meier method, and the difference between the curves was compared with the log-rank test. A Cox multivariate regression analysis was performed to assess whether age, stage and INPC could confound the results. All statistical analyses were performed with SPSS 16.0.2 for Windows and *P *values of < 0.05 were considered statistically significant.

### *In silico *screening for deregulated genes at 12q24

In order to evaluate the *in vivo *effect of the 12q24.31 gain in gene regulation, we performed *in silico *data mining of a large collection of data from the integrated microarray data resource GeneSapiens, studying its neuroblastoma and healthy samples. Briefly, GeneSapiens http://www.genesapiens.org/ is a collection of 9873 Affymetrix microarray experiments. All data is re-annotated and normalized with a custom algorithm so that all data in the database are directly comparable, with extensive biological and clinical annotation. The data is collected from various publicly available sources such as Gene Expression Omnibus and ArrayExpress. The data consists of five different Affymetrix microarray generations and covers 175 different cancers and tissue types. For a more in-depth description, see [22,23].

Gene expression levels across the data, including 308-445 healthy nervous system samples and 22-123 neuroblastoma samples, were examined. The exact number of samples available for gene expression data enquiry was dependent on the array generation that was used in the original study. We searched for a gene expression pattern showing higher expression in the neuroblastoma samples compared to the healthy nervous system samples, healthy peripheral nervous system samples, as well as healthy samples in general, indicating gene activation specifically in neuroblastoma. This was done using the Mann-Whitney-Wilcoxon rank sum method (MWW), which estimates the likelihood that the two sets of data come from the same origin. The expression patterns of the known oncogenes *MYCN*, *MEIS1 *and *ALK *were used as positive controls. The data repository accession numbers and references to the corresponding publications have been indicated in Table 1.

**Table 1:** Origin of the gene expression data used for the *in silico *analysis.

Sample set	Data repository accession number *	Number of samples	Reference to the corresponding publication
**Neuroblastoma samples**	E-MEXP-83	10	[41]
	GSE3960	22	[42]
			
**Healthy peripheral nervous system samples**	GSE1133	10	[43]
	GSE3526	8	NA
	GSE96	2	[44]
			
**Healthy central nervous system samples**	E-AFMX-2	27	[45]
	E-TABM-20	8	[46]
	GSE1133	38	[43]
	GSE1297	9	[47]
	GSE2164	87	[48]
	GSE2361	9	[49]
	GSE3526	184	NA
	GSE3960	1	[42]
	GSE5388	31	[50]
	GSE5389	11	[50]
	GSE6955	3	[51]
	GSE96	17	[44]

* Data sets with accession numbers E-MEXP-83, E-AFMX-2 and E-TABM-20 have been deposited at ArrayExpress Archive http://www.ebi.ac.uk/microarray-as/ae/, while all other data sets have been deposited at Gene Expression Omnibus http://www.ncbi.nlm.nih.gov/geo/.

## Results

### Genome-wide copy number alterations in neuroblastoma cell lines by array-CGH

**Figure 1:**
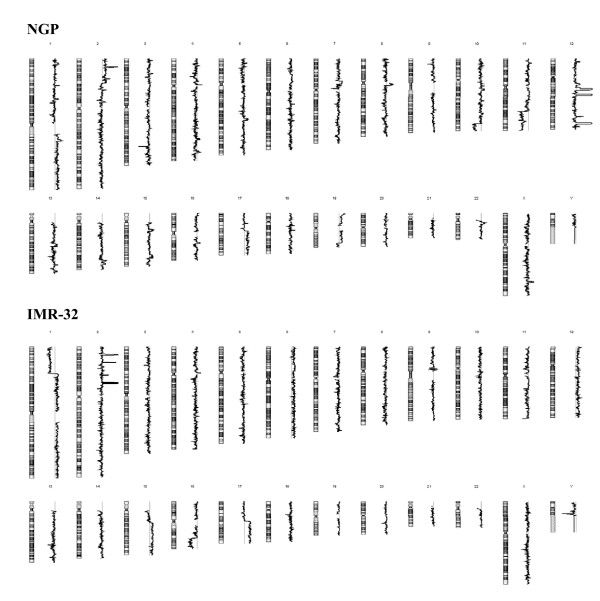
**Genome-wide copy number profiles of NGP and IMR-32 neuroblastoma cell lines**. Amplified and deleted regions are visualized as horizontal shifts to right and left of zero line, respectively.

aCGH analysis revealed several copy number changes in both cell lines (Figure 1). The most prominent copy number alterations in the IMR-32 cell line affected the short arm of chromosome 2, where three clearly distinct high-level amplifications at 2p24.3, 2p14 and 2p13.3 (14.7-16.0, 66.7-67.6, 69.1-69.3 Mb from the p-telomere) were detected, including loci for *MYCN, MEIS1 *(myeloid ecotropic viral integration site 1 homolog) and *ANTRX1*/*TEM8 *(anthrax toxin receptor 1/tumour endothelial marker 8), respectively. In addition, a high-level microamplification at 2p23 affecting only two probes for the *ALK *(anaplastic lymphoma kinase) gene was observed. Loss of copy number was detected at e.g. 1p (0-50.7 Mb) and 16q (68.3-88.7 Mb) in the IMR-32 cell line.

**Figure 2:**
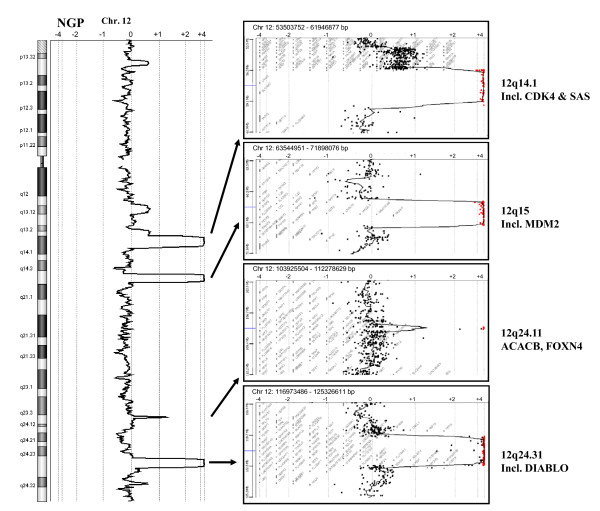
**Copy number alterations in chromosome 12 detected in the NGP cell line**. The chromosome view (left) displays the copy number profile of NGP cell line versus normal male reference. The gene view (right) magnifies the selected amplification regions at 12q14.1, 12q15, 12q24.11 and 12q24.31, indicating the log2 copy number ratios of individual oligos.

NGP cells showed high-level amplification of *MYCN *at 2p24.2-p24.3 (16.0-16.9 Mb from the p-telomere), as well as several loci at 12q, including 12q14.1, 12q15, 12q24.11 and 12q24.31 (56.4-59.4, 67.2-69.3, 108.0-108.2 and 119.9-122.4 Mb from the p-telomere) (Figure 2.). Altered regions included known targets, such as *CDK4 *(cyclin-dependent kinase 4) and *SAS *(sarcoma amplified sequence), as well as *MDM2 *(mouse double minute 2 homolog), for 12q14.1 and 12q15 amplifications, respectively. The previously unidentified microamplification at 12q24.11 involved *ACACB *(acetyl-Coenzyme A carboxylase beta) and *FOXN4 *(forkhead box N4) genes, whereas several genes were included in the detected 12q24.31 amplification. Losses of copy number were observed on chromosomes 10 (121.6-135.4 Mb), 11 (99.1-134.4 Mb) and 19 (57.0-58.9 Mb) in the NGP cell line.

### Impact of copy number alterations on gene expression

**Figure 3:**
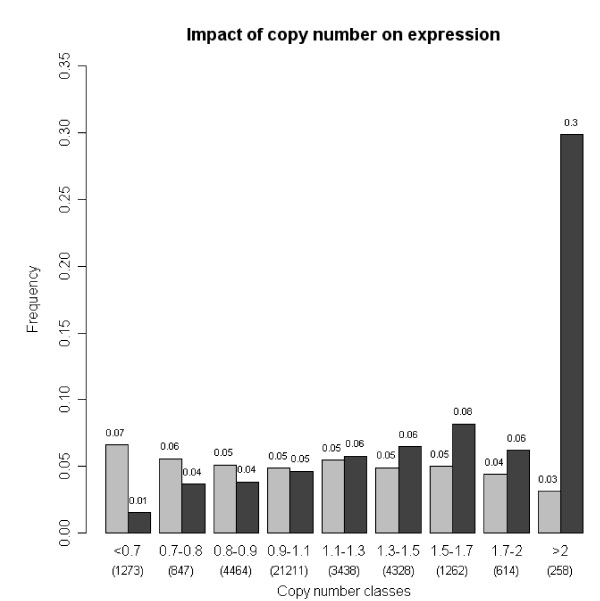
**Impact of copy number on gene expression in NGP and IMR-32 cells**. Frequency of genes (Y-axis) whose expression was high (black bars) or low (gray bars) according to copy number ratios (X-axis). High-level expression was defined as the top 7^th ^percentile of all expression ratios, while low-level expression was defined as the bottom 7^th ^percentile. The number of genes in the different copy number classes is indicated in parenthesis.

Comparison between DNA and RNA level data showed that gene expression levels across the genome were significantly influenced by copy number changes (Figure 3). The majority of the most highly amplified and overexpressed genes were located in the 2p and 12q amplicons. *MYCN *was identified as the target for 2p24 amplification in both cell lines. Other genes implicated in the IMR-32 cells in this region were *FAM84A/NSE1 *(family with sequence similarity 84, member A) and NAG (neuroblastoma-amplified protein). In IMR-32, the 2p14 amplicon showed overexpression of both *MEIS1 *and *ETAA16*, and *TEM8/ANTRX1 *was overexpressed at 2p13.3.

Genes implicated in the NGP cells at 2p24 were *MYCNOS *(*MYCN *opposite strand) and *FAM49A*. At 12q, several genes were shown to be upregulated in the distinct amplicons in the NGP cell line. In addition to the previously reported *SAS *gene, amplification and overexpresssion of e.g. *CENTG1/PIKE *(centaurin, gamma 1) and *AVIL *(advillin) were observed at 12q14.1. The 12q15 amplicon showed the characteristic involvement of *MDM2 *as well as several neighbouring genes, such as *FRS2 *(fibroblast growth factor receptor substrate 2), *CPM *(carboxypeptidase M) and *CPSF6 *(cleavage and polyadenylation specific factor 6). The 12q24.11 microamplification, which involved only *ACACB *and *FOXN4*, caused moderately increased expression of both genes in comparison to the reference sample. The most distal 12q amplicon, which is located at q24.31, included *RSN *(restin) as well as several amplified and overexpressed genes with an unknown role in neuroblastoma. Using hotspot FISH, the frequency and clinical significance of this previously poorly characterized 12q24.31 amplification in neuroblastoma was explored and compared to the frequency of 12q14-q15 amplification.

### Copy number alterations at 12q and the clinical significance of the q24 gain

**Figure 4:**
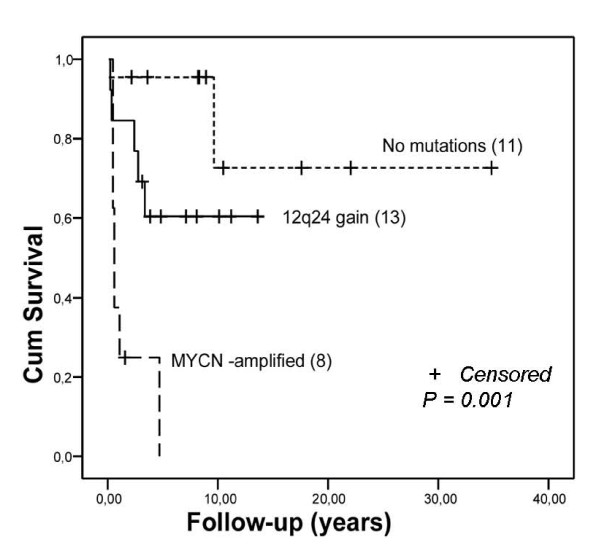
**Kaplan-Meier analysis of the survival of patients with or without *MYCN *amplification and 12q24 gain**. The prognosis of patients with 12q24 gain was intermediate when compared to those with *MYCN *amplification or patients with no amplification/gain of either locus. Numbers in parenthesis refer to the number of patients included in each category. Crosses indicate the lengths of follow-up for those patients whose follow-up was terminated before the last time point.

DNA copy number alterations at 12q14, 12q15 and 12q24.31 were analyzed using the hotspot neuroblastoma TMA format. Altogether, 2 out of 31 (6%), 6 out of 32 (19%) and 14 out of 33 (42%) informative samples showed copy number gain at 12q14, 12q15 and 12q24.31, respectively. Although high-level copy number gains of 12q24.31 were not seen in the clinical neuroblastoma samples (the mean ratio between test and centromeric probes was 1.7), a survival analysis provided evidence that low-level gain of 12q24.31 was a prognostic factor of neuroblastoma patients (*P *= 0.001) (Figure 4). Patients without *MYCN *or 12q24.31 amplification had the best prognosis, patients with the gain of 12q24.31 region had an intermediate prognosis, and patients with *MYCN*-amplified neuroblastomas had the worst prognosis (Figure 4). Interestingly, 12q24.31 gains and *MYCN *amplifications were present in different subsets of neuroblastomas. Only 1 out of 14 (7%) 12q24.31-gained neuroblastomas showed concomitant *MYCN *amplification, which was detected in altogether 7 neuroblastoma cases. Either *MYCN *amplification or 12q24.31 gain was present in 64% of all neuroblastomas. Since 12q24.31 gain did not associate with any of the other prognostic parameters, such as histology (INPC), stage, age and proliferation index (results not shown), our data suggest that it may have independent prognostic value in neuroblastoma risk stratification. A Cox multivariate regression analysis showed that age, stage and INPC did not confound the results.

### In silico screening of 12q24.31 genes in neuroblastoma and healthy nervous system samples

**Figure 5:**
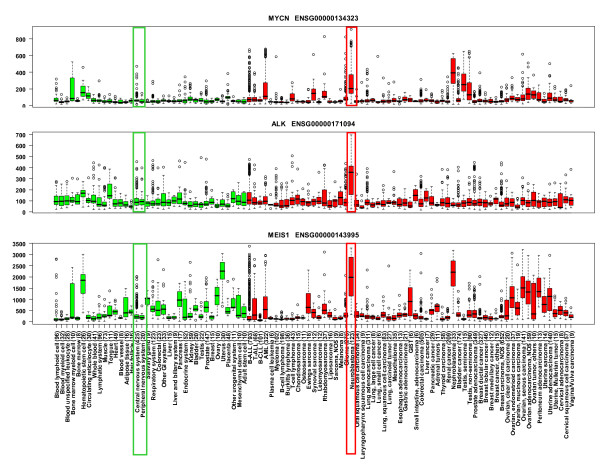
***In silico *gene expression profiles for *MYCN, ALK *and *MEIS1***. Box plot analysis of the *MYCN, ALK *and *MEIS1 *expression levels across several normal (green boxes) and cancer tissues (red boxes). The number of samples included in each tissue type is indicated in parenthesis. Normal central nervous system and peripheral nervous system samples, as well as neuroblastoma samples are highlighted with green and red rectangles, respectively. The expression levels of all three control genes are highly elevated in neuroblastoma.

**Figure 6:**
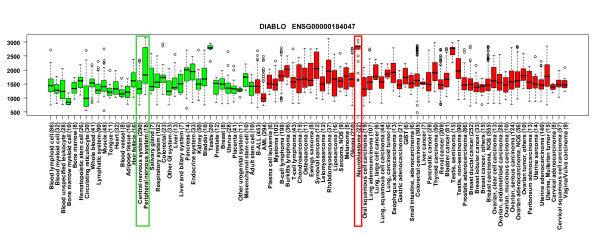
***In silico *gene expression data suggest a role for *DIABLO *in neuroblastoma**. Box plot analysis of the *DIABLO *expression levels across normal (green boxes) and cancer tissues (red boxes). The number of samples included in each tissue type is indicated in parenthesis. Normal central nervous system and peripheral nervous system samples, as well as neuroblastoma samples are highlighted with green and red rectangles, respectively. *DIABLO *shows a clear activation in a subset of neuroblastoma samples.

The expression levels of the control genes *(MYCN, MEIS1 *and *ALK*) were, in all three cases, when compared to all healthy samples, healthy nervous system samples and healthy peripheral nervous system samples, highly and statistically significantly elevated in neuroblastoma (Table 2, Figure 5). Of the 40 genes in the 12q24.31 amplicon, 25 (62.5%) had data in GeneSapiens. Among these 25 informative genes, five genes had statistically elevated expression in neuroblastoma when compared to healthy tissue samples, healthy nervous system and healthy peripheral nervous system samples. These genes were *DIABLO *(diablo homolog, Drosophila), *ZCCHC8 *(zinc finger, CCHC domain containing 8), *RSRC2 *(arginine/serine-rich coiled-coil 2), *KNTC1 *(kinetochore associated 1) and *MPHOSPH9 *(M-phase phosphoprotein 9) (Table 2). Among these, *DIABLO *showed the strongest and most specific activation in a subset of neuroblastoma samples (Figure 6, see Additional files 1, 2, 3, and 4).

**Table 2:** Analysis of *in silico *gene expression data with Mann-Whitney-Wilcoxon rank sum method (MWW) revealed elevated expression of five genes at 12q24.31 amplicon.

Gene name/Genomic location	P-value for test that expression is higher in neuroblastoma than in		
	healthy nervous system samples	healthy peripheral nervous system samples	all healthy tissue samples
***MYCN*/2p24.1**	<1e-16	6.2e-11	<1e-16
***ALK*/2p23**	<1e-16	3.6e-7	<1e-16
***MEIS1*/2p14-p13**	<1e-16	9.64e-13	<1e-16
***DIABLO*/12q24.31**	<1e-16	0.00302	<1e-16
***ZCCHC8*/12q24.31**	<1e-16	<1e-16	1e-05
***RSRC2*/12q24.31**	<1e-16	0.00031	<1e-16
***KNTC1*/12q24.31**	<1e-16	<1e-16	<1e-16
***MPHOSPH9*/12q24.31**	<1e-16	<1e-16	<1e-16

Gene expression levels between 22-123 neuroblastoma samples and healthy tissue samples, including 308-445 healthy nervous system (including central and peripheral nervous system samples) and 18-20 peripheral nervous system samples, were examined. In addition to the 12q24.31 amplicon genes, *MYCN, ALK *and *MEIS1 *were included in the analysis as controls. The analysis showed statistically elevated expression of *DIABLO, ZCCHC8, RSRC2, KNTC1 *and *MPHOSPH9 *in neuroblastoma samples (*P *< 0.01).

## Discussion

Our integrative microarray-based analysis of gene copy number and gene expression in two neuroblastoma cell lines characterized many previously known genetic aberrations, and revealed a previously poorly characterized amplification of 12q24.31 in neuroblastoma. The significance of the detected 12q24.31 amplicon in clinical samples was further validated using the hotspot neuroblastoma TMA format, which revealed low-level gain of 12q24.31 in 42% of neuroblastoma patients. Patients with 12q24.31 gain had an intermediate prognosis in comparison to patients with *MYCN *amplification (poor prognosis) and patients having neither of the two alterations (best prognosis) (*P *= 0.001). Moreover, although the NGP cell line was shown to contain both *MYCN *and 12q24.31 amplifications, we found only one neuroblastoma in the tissue array analysis with both genetic modifications. Whether the effects of the 12q24.31 gain and *MYCN *amplification represent actually two alternative routes in neuroblastoma progression, remains, however, to be investigated further in separate studies.

As neuroblastomas display vast intratumoral heterogeneity of various biological variables, including *MYCN *copy number differences between cells [24], an overall estimation of the prognostic impact of copy number changes and other genetic alterations in one randomly selected area of the tumor may be erroneous. Especially low-level copy number increases of prognostically significant genes or genomic regions are difficult to detect. Moreover, since cancer cell lines often originate from a single clone, the results of *in vitro *studies with cancer cells lines are not necessarily applicable to intratumorally heterogeneous tumors, such as neuroblastoma. Therefore, on the basis that accelerated cell proliferation is associated with progression-related chromosomal changes, we measured the copy number of the 12q24.31 region in the most proliferative regions of neuroblastoma samples, *i.e*. hotspots [20]. With this sensitive methodology, low-level gain of 12q24.31 was shown to occur frequently and define a biologically distinct subpopulation of patients with intermediate outcome.

Supportive evidence of the 12q24 gain in neuroblastoma has recently been reported by Mosse and co-authors [25]. In their BAC-array-based CGH study the authors report 32% of the analyzed 82 primary tumors to show low-level gain of 12q24 with minimal common region between 110.8-132.4 Mb from the p-telomere. The reported frequency resembles closely to that detected in our hotspot FISH analysis (42%). Moreover, Mosse and co-authors identified the low-level gain of 12q24 to correlate with aggressive clinical phenotype in a subset of tumors without MYCN amplification. These data are in excellent concordance with the data presented here.

In order to define activated genes within the detected subtle copy number increase, we performed an *in silico *analysis of vast amount of existing gene expression data [22], including a considerable collection of normal peripheral nervous system and neuroblastoma samples. Using this approach we identified elevated expression of five genes located at the 12q24.31 amplicon; *DIABLO, ZCCHC8, RSRC2, KNTC1 *and *MPHOSPH9*. Among these five genes, *DIABLO *showed substantially elevated expression levels in a subset of the neuroblastoma samples, suggesting that it may have an oncogenic role in these tumors. DIABLO has been reported to have proapoptotic effects in cells and therefore overexpression of *DIABLO *is believed to cause antitumoral activity via cancer cell sensitization to apoptotic cell death [26,27]. Even though the exact role of *DIABLO *in neuroblastoma remains to be elucidated, previous contradictory results suggest that *DIABLO *has both a direct and inverse correlation to prognosis in various cancers. In cervical and gastric cancer, *DIABLO *activation has been shown to associate with unfavourable prognostic parameters [28-30]. On the contrary, in renal cell carcinoma, lung cancer and hepatocellular carcinoma, *DIABLO *expression associates with favourable prognostic factors [31-33]. In this sense, the carcinogenetic role of *DIABLO *is ambiguous, and needs to be examined more thoroughly, also in neuroblastoma.

In addition to *DIABLO*, also *ZCCHC8, RSRC2, KNTC1 *and *MPHOSPH9 *showed statistically increased expression in neuroblastoma samples when compared to the groups of healthy samples. Although the increase in expression levels of these genes were marginal, which could be argued to be due to impact of the detected low-level gain, and the activation was not neuroblastoma specific, these genes may also have a role in neuroblastoma. Moreover, as only 63% of the genes within the detected amplicon were informative in the *in silico *analysis, we cannot rule out the possibility that additional oncogenic genes may reside at 12q24.31.

Data integration at the DNA and RNA level provided evidence that several previously known oncogenes were activated due to DNA amplification at corresponding sites. These previously known oncogenes included *MYCN *(in the IMR-32 and NGP cell lines), as well as *ALK, MEIS1 *and *TEM8 *(in the IMR-32 cell line) [34-37], which all located at 2p. In addition to oncogene activations at 2p, distinct high-level amplification sites were mapped at high-resolution along chromosome 12 (at q14, q15, q24.11 and q24.31) in the NGP cell line. In addition to the genes previously implicated in the 12q14 and q15 amplicons, such as *SAS *and *MDM2*, we found amplification and overexpression of several other genes, including *CENTG1 *and *AVIL *at 12q14, and *FRS2, CPM *and *CPSF6 *at 12q15. In addition to *DIABLO*, our data revealed several unreported, amplified and overexpressed genes in the 12q24.31 region, locus from which only restin (*CLIP1/RSN*) has been previously shown to be amplified in NGP cells [38]. Within the limits of this study, the detected gene amplifications may serve as additional data in future research on prognostically significant genetic alterations in neuroblastoma.

Recently, few large studies with high-resolution genome-wide copy number analysis have been published [25,39,40]. Some of these studies include, similarly to this study, parallel gene expression analysis allowing the identification of concomitant alterations in copy number and expression level [25,39]. The obvious strength of these studies is the vast number of samples included in the analyses. Although the study presented here is limited by the sample size in the initial aberration discovery phase, we show that the utilization of only a few representative model systems can, nevertheless, be used to identify clinically relevant data. Furthermore, the *in silico *data mining approach used here for evaluating transcriptional consequences of the detected aberration shows the power of utilization of previously published gene expression data providing a rapid and cost-effective tool for the discovery of new biomarkers.

## Conclusions

Our results illustrate that the integration of array-based profiling at DNA, RNA and tissue levels is a powerful strategy in the identification of amplified genes with simultaneous overexpression, and highlights the significance of *in silico *data mining opportunities in providing further evidence of putative activated genes involved in cancer progression. In addition to confirming previously identified oncogenes in neuroblastoma, our analysis led to the identification of 12q24.31 gain as a novel marker in neuroblastoma progression, as well as upregulation of *DIABLO *specifically in a subset of neuroblastoma samples within this region. Clearly, extensive functional and clinical investigations are needed to understand the complete role of this apoptosis-related protein in neuroblastoma pathobiology before its potential as a therapeutic target or predictor of outcome can be determined.

## Competing interests

The authors declare that they have no competing interests.

## Authors' contributions

MW participated in the design of the study, carried out the aCGH and gene expression profiling, participated in the *in silico *data analysis and wrote the manuscript. MK carried out the statistical analysis of the FISH data, provided the MYCN status of the tissue samples, and revised the manuscript. RK carried out the FISH analysis. HE performed normalization of the gene expression data and integration of expression and aCGH data. SK and KO collected the in silico gene expression data used for this study and performed the in silico data analysis. AK participated in the design of the study and revised the manuscript. SM participated in the design of the study and revised the manuscript. HH carried out histopathological analysis of the neuroblastoma samples. All authors read and approved the final manuscript.

## Pre-publication history

The pre-publication history for this paper can be accessed here:

http://www.biomedcentral.com/1471-2407/10/181/prepub

## Supplementary Material

Additional file 1 ***In silico *****gene expression profile for *ZCCHC8*.** Click here for file

Additional file 2 ***In silico *****gene expression profile for*****RSRC2***. Click here for file

Additional file 3 ***In silico *****gene expression profile for *****KNTC1***. Click here for file

Additional file 4 ***In silico *****gene expression profile for *****MPHOSPH9***. Click here for file
