# Supplementary figures and images for: Array-based gene expression, CGH and tissue data defines a 12q24 gain in neuroblastic tumors with prognostic implication
Source: BMC Cancer. 2010 May 5;10:181. doi: 10.1186/1471-2407-10-181 (PMC2873396; doi:10.1186/1471-2407-10-181)

ZCCHC8 ENSG00000033030

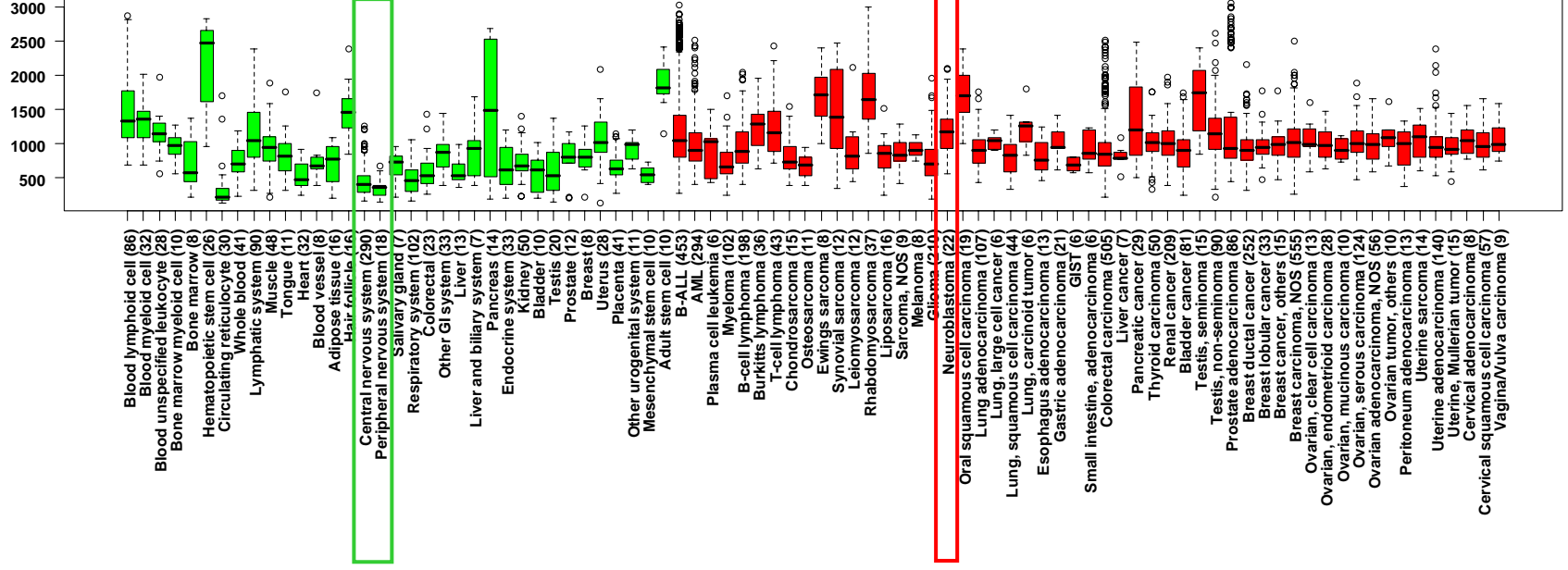

Supplement: Additional file 1 — In silico gene expression profile for ZCCHC8. [file 1471-2407-10-181-S1.PDF]

RSRC2 ENSG00000111011

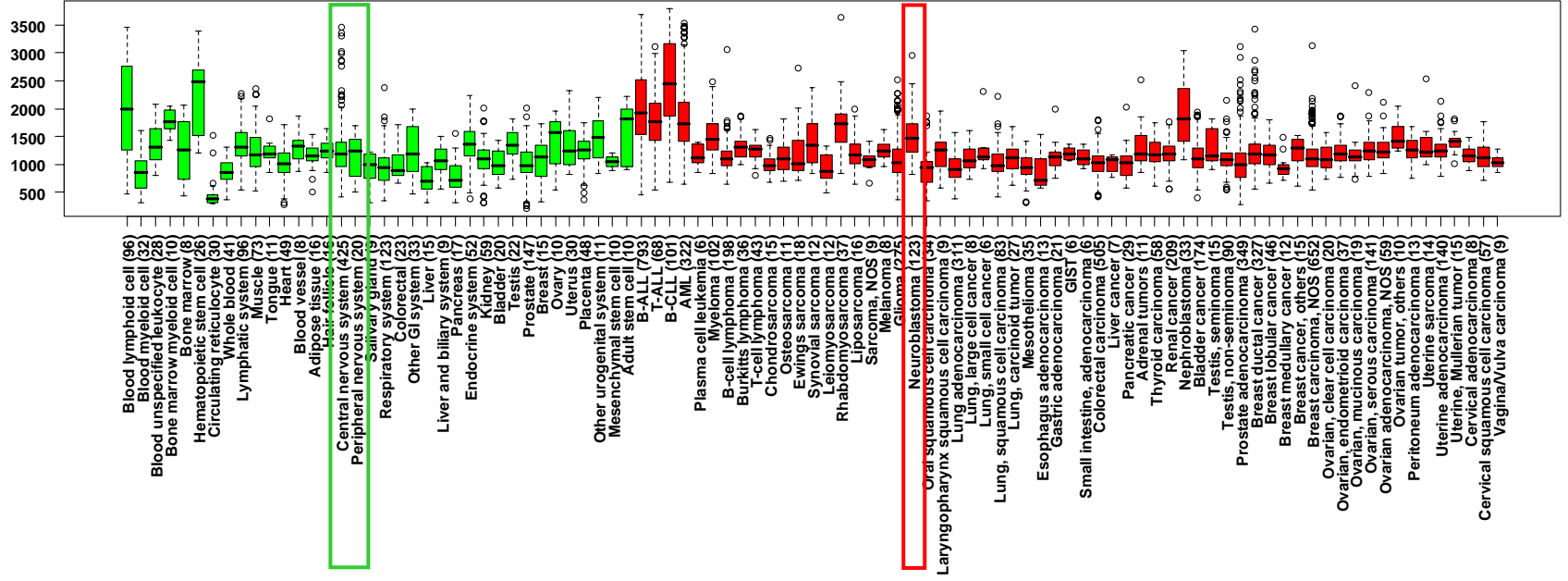

Supplement: Additional file 2 — In silico gene expression profile forRSRC2. [file 1471-2407-10-181-S2.PDF]

KNTC1 ENSG00000184445

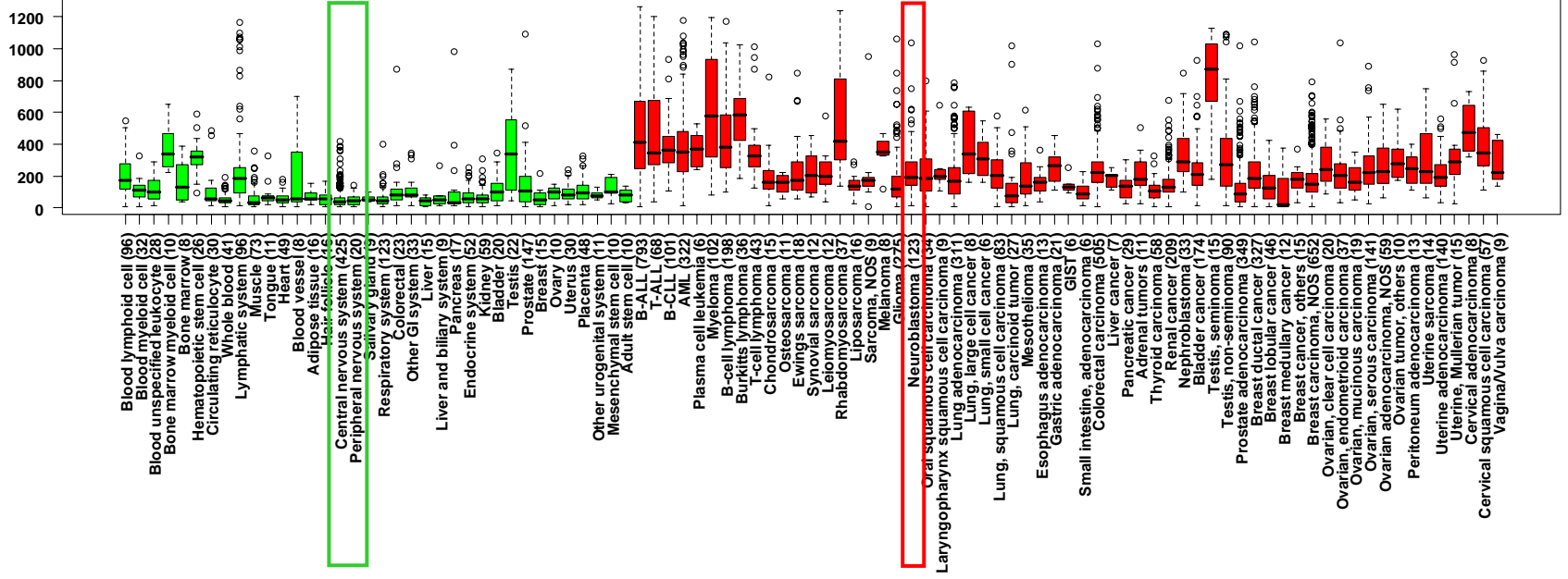

Supplement: Additional file 3 — In silico gene expression profile for KNTC1. [file 1471-2407-10-181-S3.PDF]

MPHOSPH9 ENSG00000051825

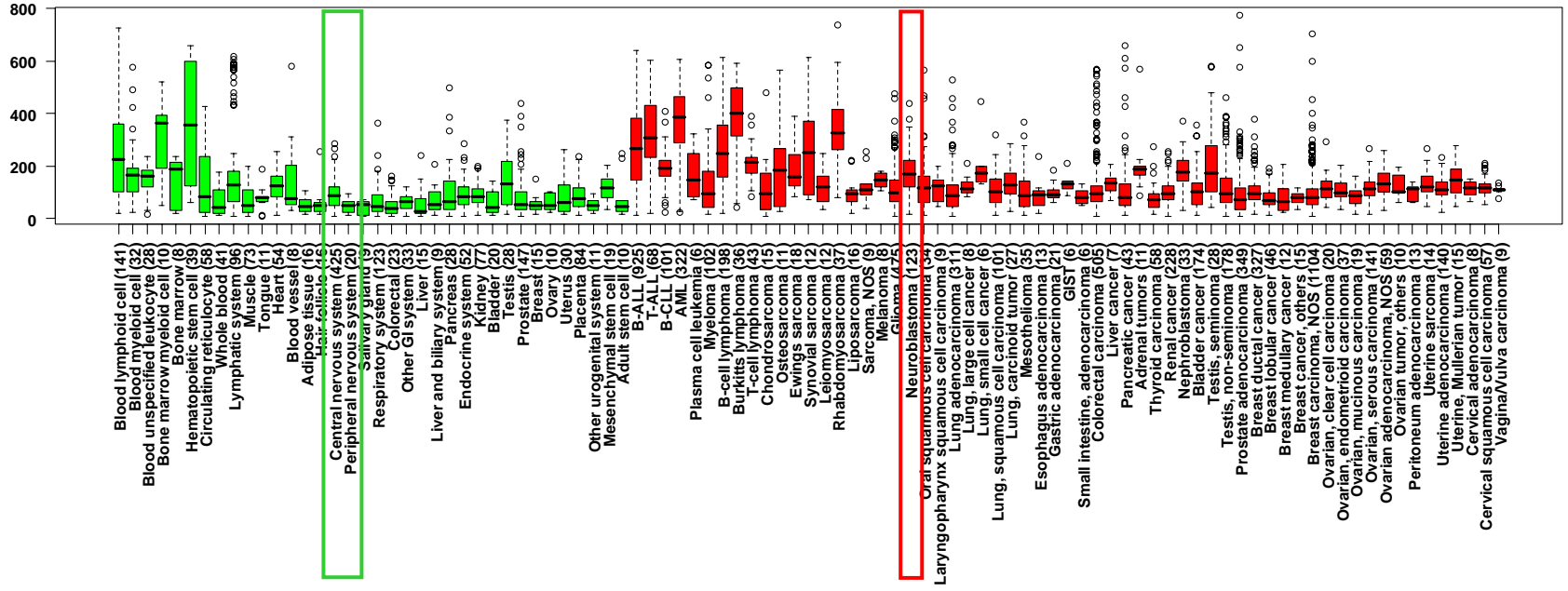

Supplement: Additional file 4 — In silico gene expression profile for MPHOSPH9. [file 1471-2407-10-181-S4.PDF]
